# Supplementary material for: Identification of distinct functional thymic programming of fetal and pediatric human γδ thymocytes via single-cell analysis
Source: Nat Commun. 2022 Oct 4;13:5842. doi: 10.1038/s41467-022-33488-2 (PMC9532436; doi:10.1038/s41467-022-33488-2)
Supplement: Supplementary file 1 — Supplementary Information [file 41467_2022_33488_MOESM1_ESM.pdf]

A

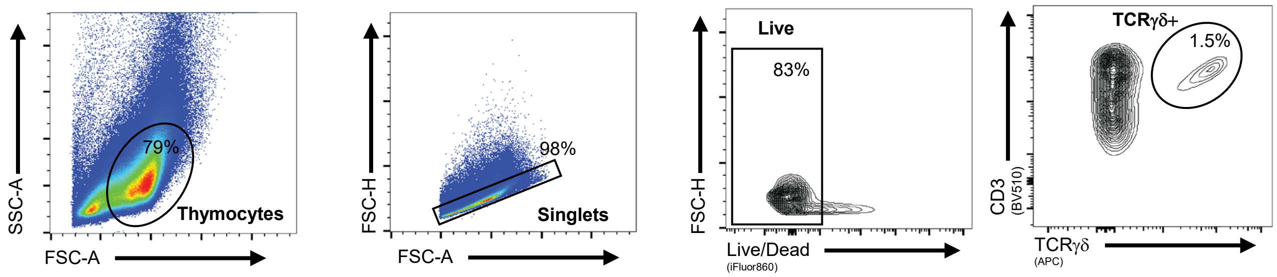

B

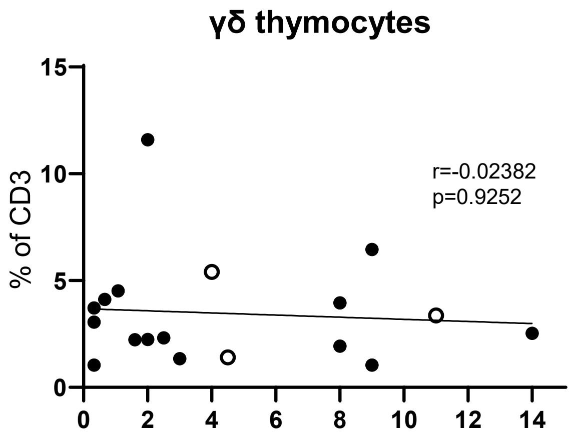

Supplementary Figure 1. Flow data from the human γδ thymocyte samples used in the sc RNA/TCR-seq experiments. 6 fetal and 3 pediatric thymus samples were processed and stained (as described in Methods section) in 3 independent experiments. **(A)** Representative FACS gating used to sort Live CD3+ γδ+ thymocytes prior to perform 10x genomics Chromium Single Cell V(D)J protocol. **(B)** Frequency (%) of pediatric γδ thymocytes out of total CD3+ live cells. White dots indicate samples whose cells were sorted to generate sc RNA/TCR-seq libraries. R and p values (two-tailed) in **(B)** were obtained by using nonparametric Spearman correlation. Source data are provided as a Source Data file.

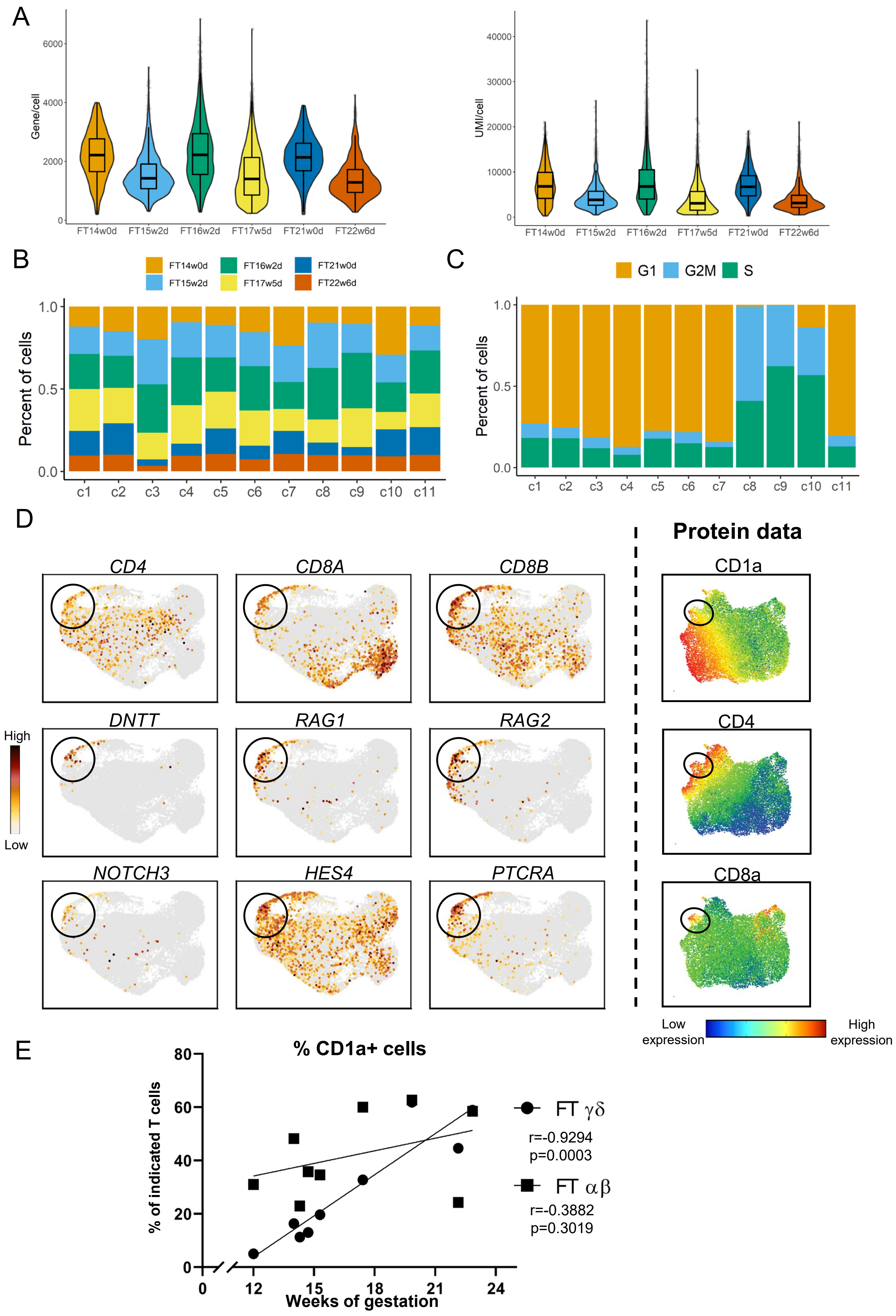

Supplementary Figure 2. (legend: see next page)

Supplementary Figure 2. Transcriptomic and proteomic data related to fetal thymus. (A) Quality control (QC) plots of the six fetal thymus sc RNA-seq libraries. **(A, left)** Detected gene number per cell. **(A, right)** Mean number of Unique Molecular Identifier (UMI) per cell. The three horizontal lines of the box–whisker plot represent the higher quartile, median, and lower quartile, respectively. The whiskers stretch from each quartile to the maximum or minimum. **(B)** Bar plot displaying individual subject cell distribution across clusters identified in Fig. 2A. **(C)** Bar plot displaying the proportions of cells classified according to their cell cycle phase in each cluster identified in Fig. 2A. **(D)** Transcript expression levels (left) or protein expression levels (right) of markers which allow the identification of a highly proliferative and actively rearranging cell subset (identified by black circles) described previously (van Coppernolle, Leukemia, 2012). **(E)** Dot plot displaying abundance of CD1a+ cell populations across gestation time in the αβ (squares) and γδ T (circles) cell lineages. Data was obtained from flow cytometry experiments (n=9 biologically independent fetal thymuses). R and p values (two-tailed) were determined by Spearman correlation. Source data are provided as a Source Data file.

A

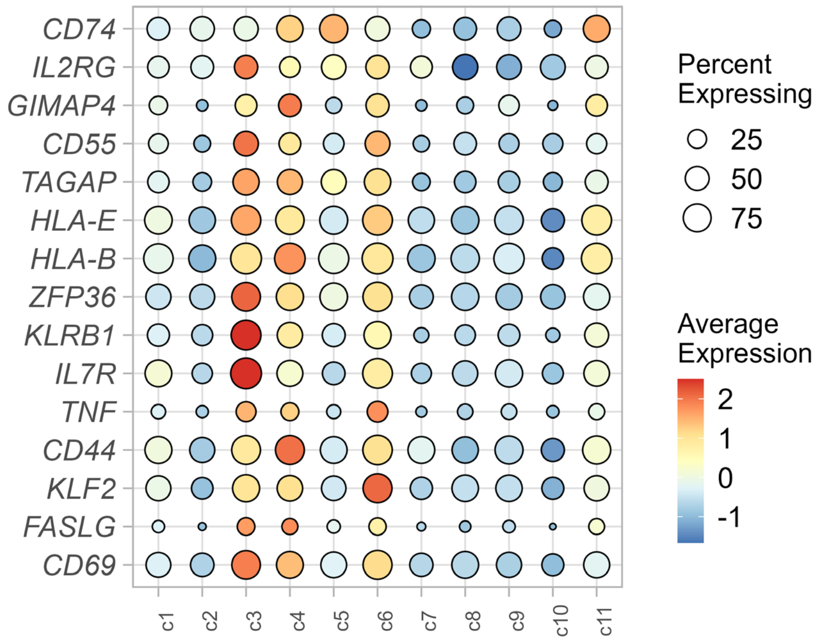

B

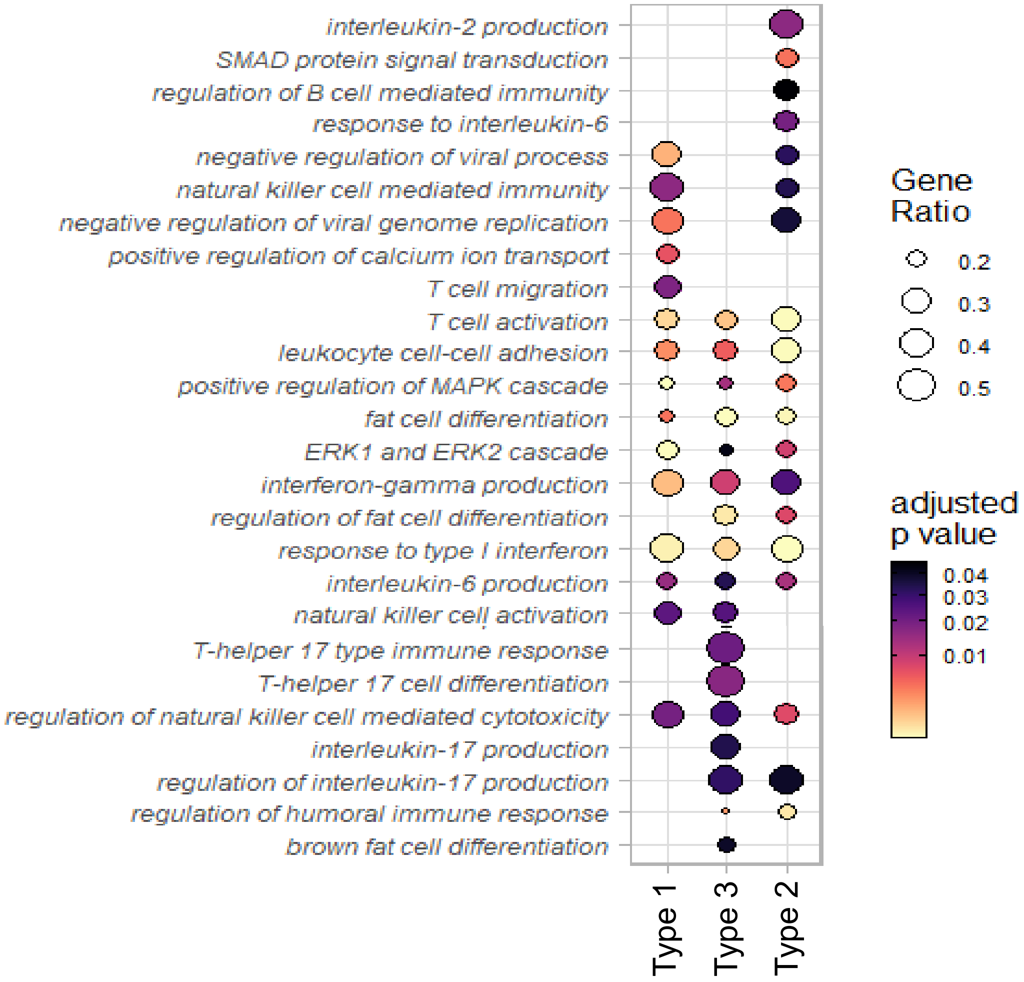

Supplementary Figure 3. (legend: see next page)

Supplementary Figure 3. Characterization of type 1, type 3 and type 2-like effector clusters of the fetal thymus. (A) Dot plot heatmap displaying row-scaled Log2 fold change (logFC) expression values of selected differentially expressed genes (DEGs) by c3, c4 and c6 clusters (Type 1, Type 3 and Type 2-like from **Fig. 3B**) after comparing them against the other cell clusters. The size of the dots represents the percentage of cells in the cluster expressing the feature while color indicates average logFC expression. The complete list of markers is provided in **Supplementary Data 3. (B)** Dotplot heatmap showing GO biological process terms enriched after performing Gene Set Enrichment Analysis (GSEA) on the different effector clusters. Size of the dots represent gene ratio (number of genes that belong to a given gene-set divided by the total size of the gene-set), while color illustrates the level of the p adjusted values (Benjamin-Hockberg correction). P adjusted value color is represented as a square-root based scale.

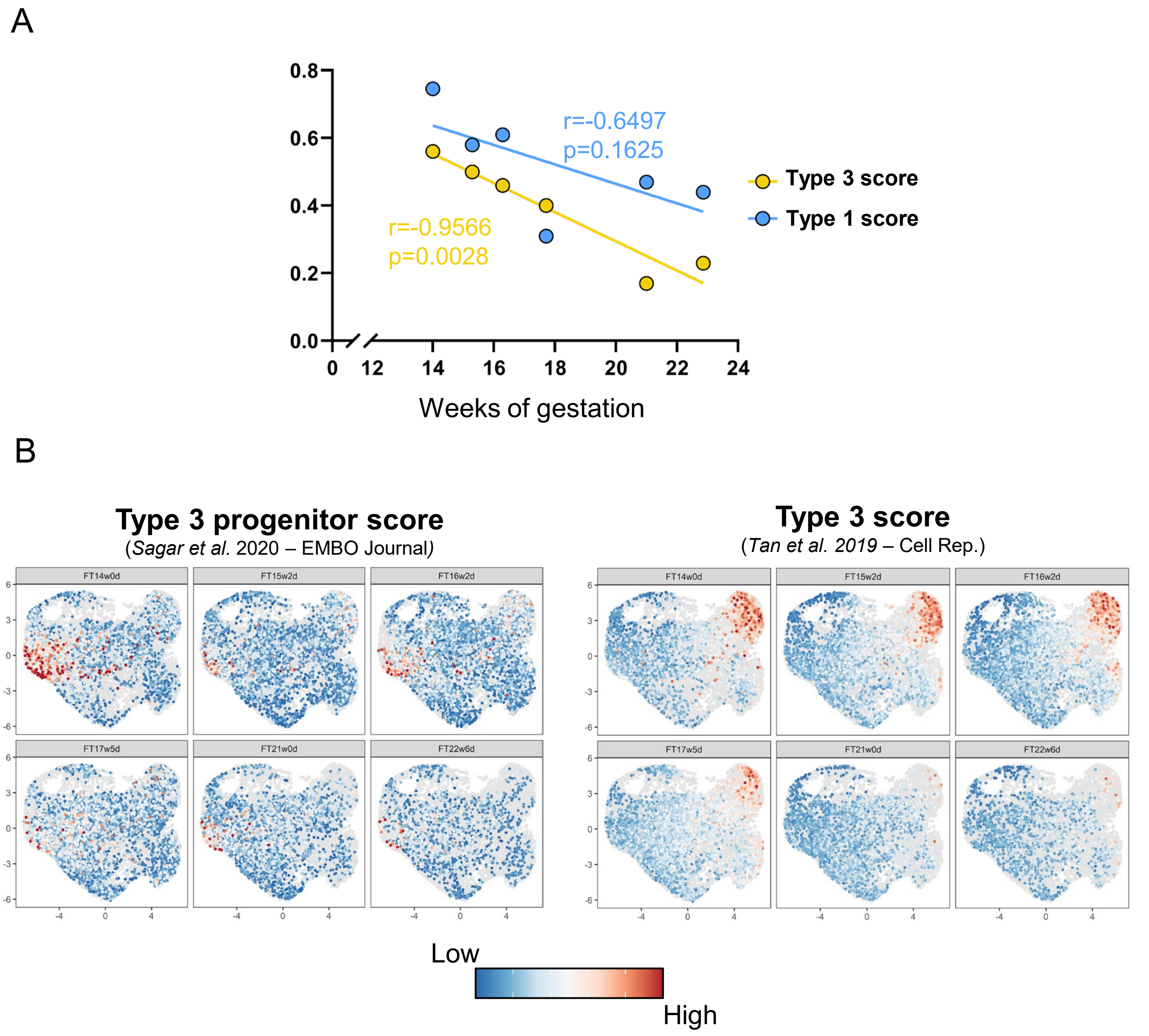

Supplementary Figure 4. Wave-like pattern of type 3 programming on fetal γδ thymocytes. (A) Dot plot indicating mean values of Type 1 or Type 3 score across gestation time in Type 1 (c4) or Type 3 (c3) clusters respectively of the fetal thymus sc data set (n=6). **(B)** UMAP plots displaying the Type 3 progenitor score and Type 3 score splitted by subject. The scores were computed using different set of markers described in (**Supplementary Data 2**); each cell is colored based on its individual score. R and p values (two-tailed) in (A) were obtained by using Spearman correlation. Source data are provided as a Source Data file.

A

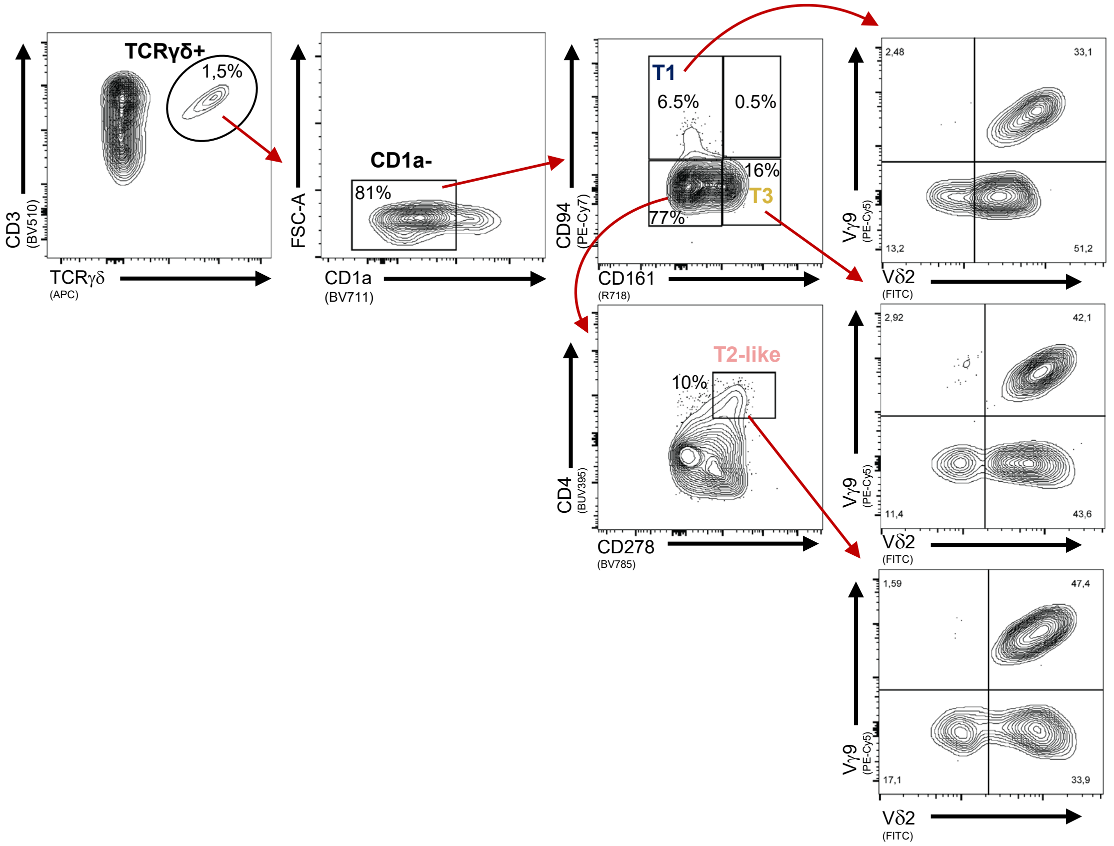

B

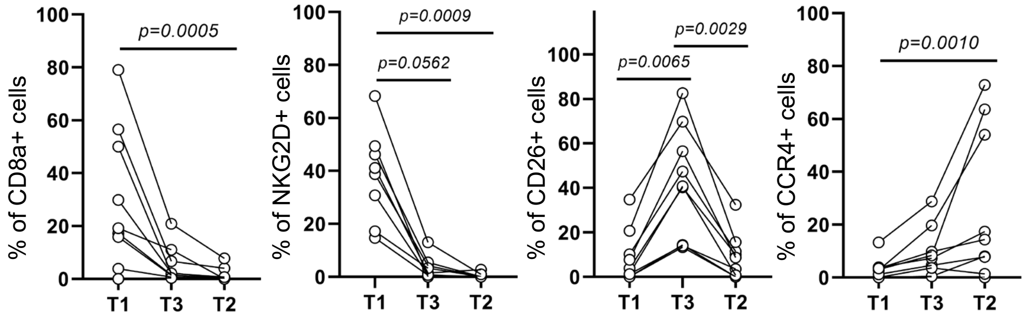

Supplementary Figure 5. Protein validation by flow cytometry of the distinct fetal γδ effector clusters. (A) Representative gating strategy used to identify type 1 (T1), type 3 (T3) and type 2-like cells. γδ+ effector cells were gated as CD1a-CD94+CD161- (Type 1), CD1a-CD94-CD161$_{hi}$+CD26+ (Type 3) and CD1a-CD94-CD161-/$_{int}$CD4+ICOS+ (Type 2-like). Cells were further sub-gated to assess the abundance of the distinct γδ subsets in each effector population using Vδ2 and Vγ9 TCR markers. **(B)** Paired dot plots representing the abundance of CD8a+, NKG2D+, CD26+ and CCR4+ cells in the distinct effector clusters. Data in **(A,B)** from 8 fetal thymus samples. **(B)** was analyzed by Ordinary one-way ANOVA for matched data with Holm-Sidak's multiple comparisons test as Post Hoc test when data followed normality or Friedman test followed by Dunn's test as Post Hoc test when data was not following normality. Source data are provided as a Source Data file.

A

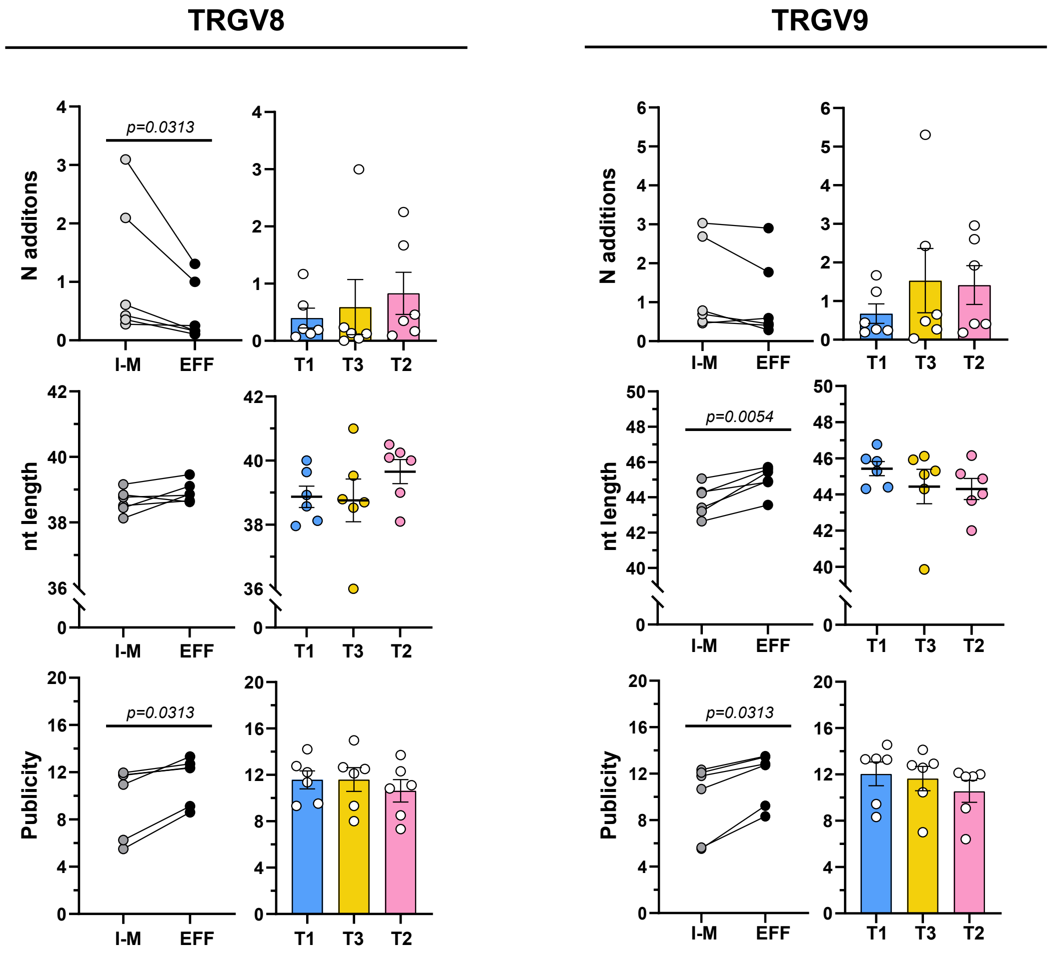

B

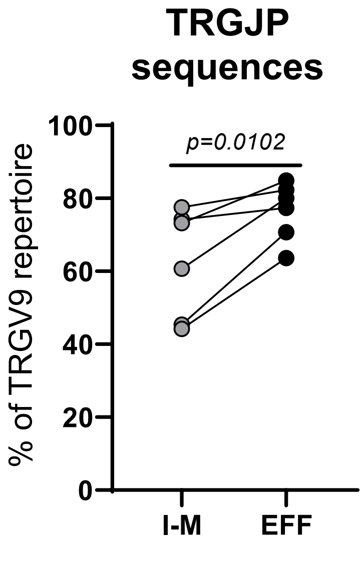

Supplementary Figure 6. (legend: see next page)

Supplementary Figure 6. Fetal sc γδ thymocyte TRG CDR3 data. **(A)** Mean levels of N additions (top), CDR3 nt length (middle) and publicity levels (bottom) of fetal γδ thymocytes with TRGV8-containing (left column) and fetal TRGV9-containing (right column) CDR3 sequences (n=6 fetal thymus samples). More data related to TRGV usage can be found in **Fig.5A. (B)** Paired dot plot indicating the frequency (%) of TRGV9-TRGJP sequences in the TRGV9 repertoire (n=6 fetal thymus samples). Error bars in **(A)** correspond to the SEM. I-M and EFF values were compared by Wilcoxon matched-pairs signed rank test, while T1,T2 and T3 groups were compared by Ordinary one-way ANOVA with Tukey's multiple comparisons test as Post Hoc test. “I-M” group: immature/maturing. “EFF” group: “effector”. “T1”,”T3” and “T2” groups: Type 1, Type 3 and Type 2-like. Source data are provided as a Source Data file.

A

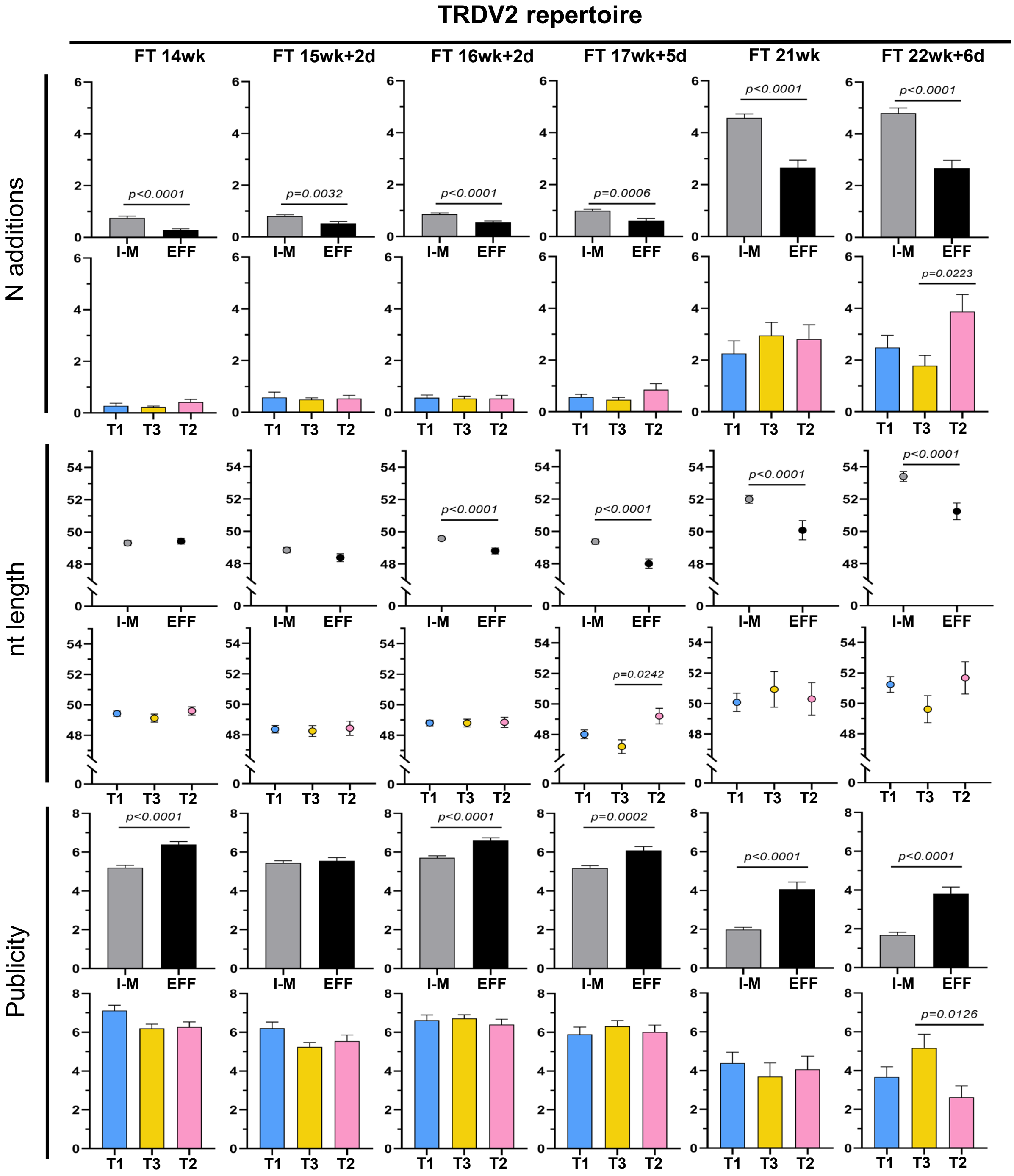

B

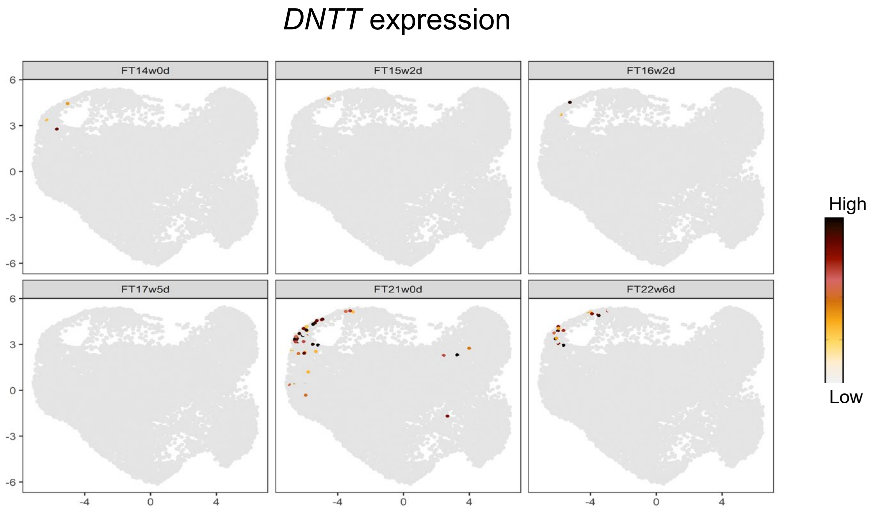

Supplementary Figure 7. (legend: see next page).

Supplementary Figure 7. Data connected to fetal sc γδ thymocyte CDR3 data. (A) Plots displaying mean number of N additions (top), nt length (middle) and publicity levels (bottom) from TRDV2 CDR3 sequences divided by subject (n=6). **(B)** *DNTT* (TdT enzyme) expression levels in the UMAP representation and splitted by fetal subject. In **(A)** Bar plots/Dot plots error bars correspond to the SEM. I-M and EFF values were compared by Mann-Whitney test, while T1,T2 and T3 groups were compared by Kruskal-Wallis test with Dunn's multiple comparisons test as Post Hoc test. "I-M" group: immature/maturing. "EFF" group: "effector". "T1","T3" and "T2" groups: Type 1, Type 3 and Type 2-like.

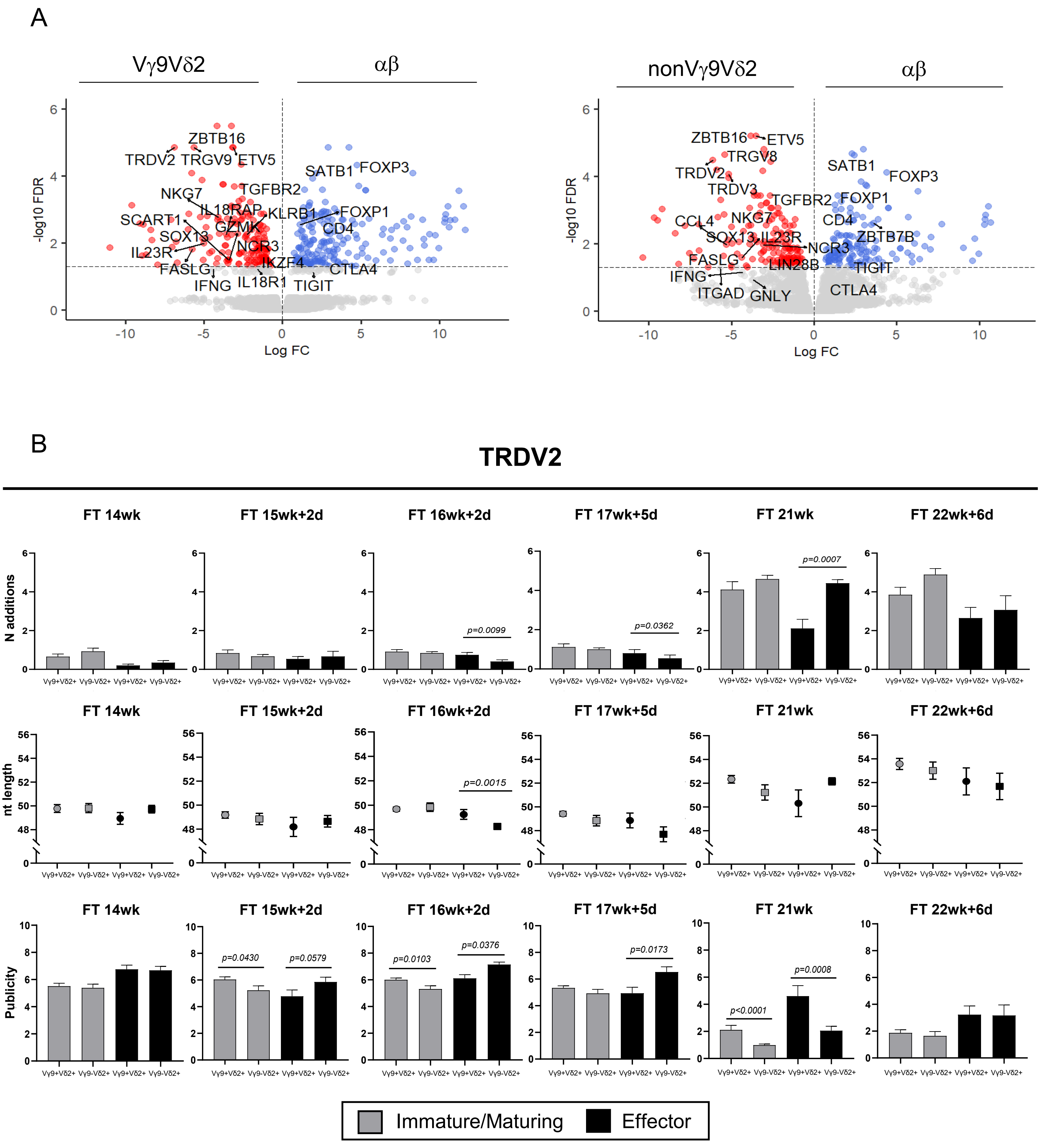

Supplementary Figure 8. (legend: see next page).

Supplementary Figure 8. Fetal Vγ9Vδ2 and nonVγ9Vδ2 thymocytes show similar effector programming and public CDR3 features. (A) Volcano plot illustrating differentially expressed genes obtained from RNA-seq data after comparing Vγ9Vδ2 or nonVγ9Vδ2 and αβ thymocytes. Cell populations were sorted from different subjects than the ones used in the sc experiment (n=3, age= 17 wk, 17 wk and 19wk of gestation). **(B)** Bar plots and dot plots indicating mean number of N additions (top), mean CDR3 nt length (middle) and mean level of CDR3 publicity (bottom) from TRDV2-CDR3 containing cells from either Vγ9Vδ2 or nonVγ9Vδ2 cell populations splitted by subject (n=6) as identified in the sc TCR seq data. In **(B)** Bar plots/Dot plots are means ± SEM. Comparison between Vγ9+Vδ2+ and Vγ9+Vδ2- cells was performed by Kruskal-Wallis test with Dunn's multiple comparisons test as Post Hoc test. "I-M" group: immature/maturing. "EFF" group: "effector".

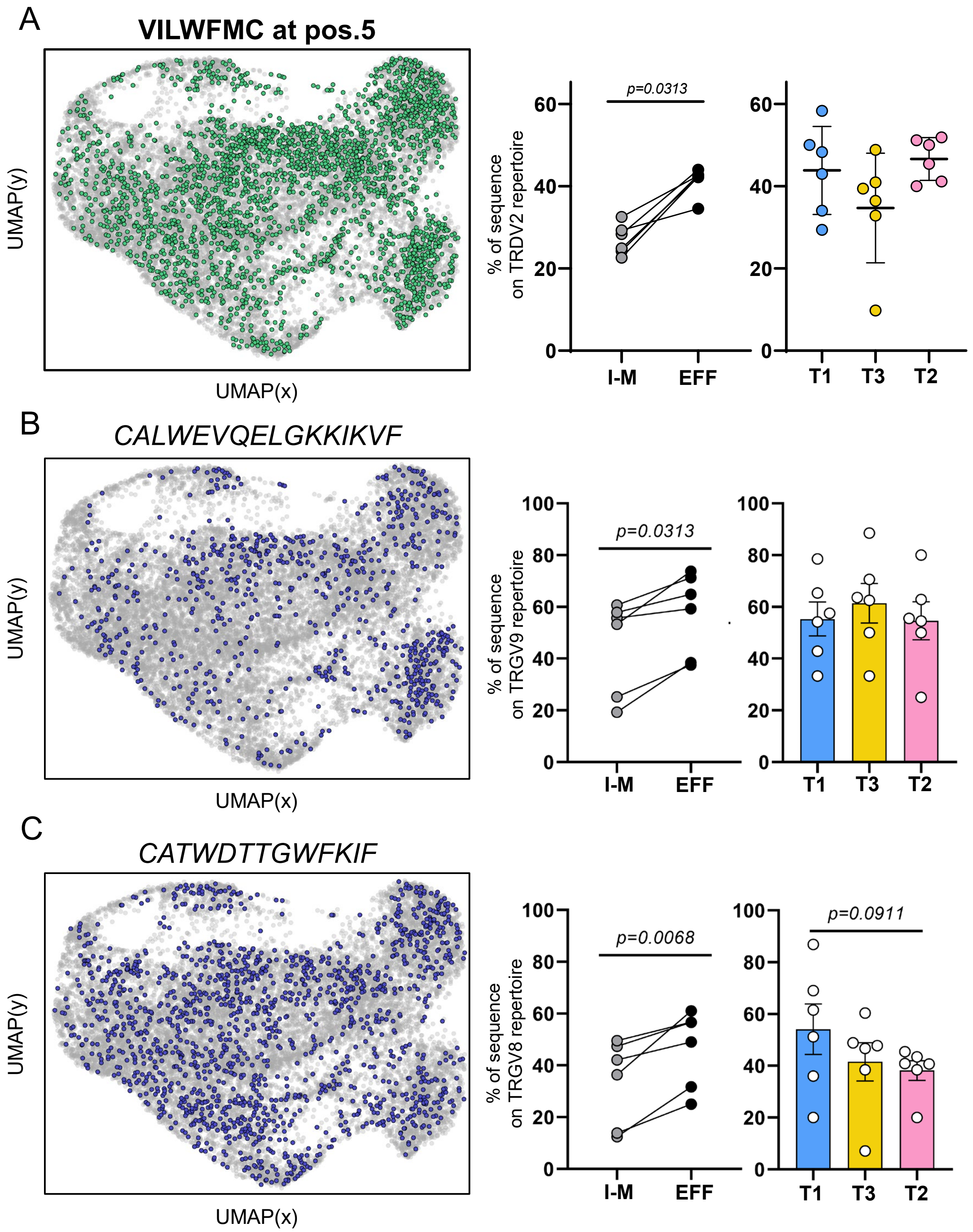

Supplementary Figure 9. Type 1, type 3 and type 2 γδ thymocytes display similarities in their CDR3γ sequences. **(A)** Plots indicate distribution (UMAP; left) and percentage (center and right) of cells with a TRDV2-containing CDR3 sequence with a highly hydrophobic residue (V, I, L, W, F, M or C) at position 5. **(B)** Plots indicate distribution (UMAP; left) and percentage (center and right) of cells containing a CALWEVQELGKKIKVF TRGV9 sequence. **(C)** Plots indicate distribution (UMAP; left) and percentage (center and right) of cells containing a CATWDTTGWFKIF TRGV8 sequence. Light grey dots in the UMAPS indicate cells without a highly hydrophobic residue at position 5 of the CDR3δ region **(A)** or with a CDR3γ sequences different than the highlighted ones or cells without detected CDR3γ sequence **(B,C)**. Error bars in **(A,B,C)** correspond to the SEM. I-M vs EFF comparision in **(A,B,C)** was analyzed with two-tailed paired t-test, while comparisions between effector clusters were performed by Ordinary one-way ANOVA for matched data with Holm-Sidak's multiple comparisons test as Post Hoc test (n=6 fetal thymus samples). "I-M" group: immature/maturing. "EFF" group: "effector". "T1","T3" and "T2" groups: Type 1, Type 3 and Type 2-like. Source data are provided as a Source Data file.

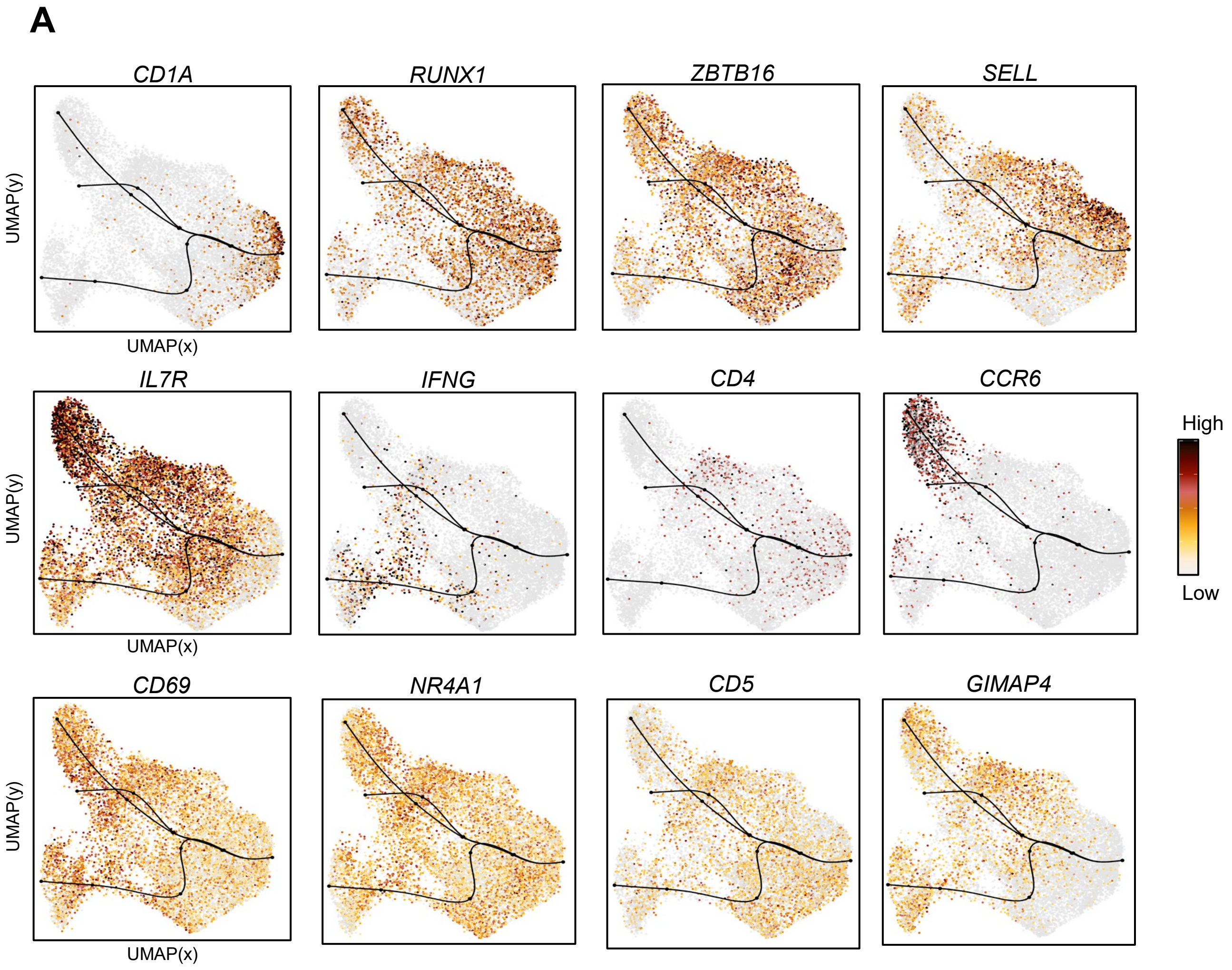

Supplementary Figure 10. Genes changing across the distinct developmental pathways identified for the fetal γδ effector clusters. Expression levels of selected genes in the fetal γδ thymocyte UMAP (n=6 subjects) combined with Slingshot trajectories.

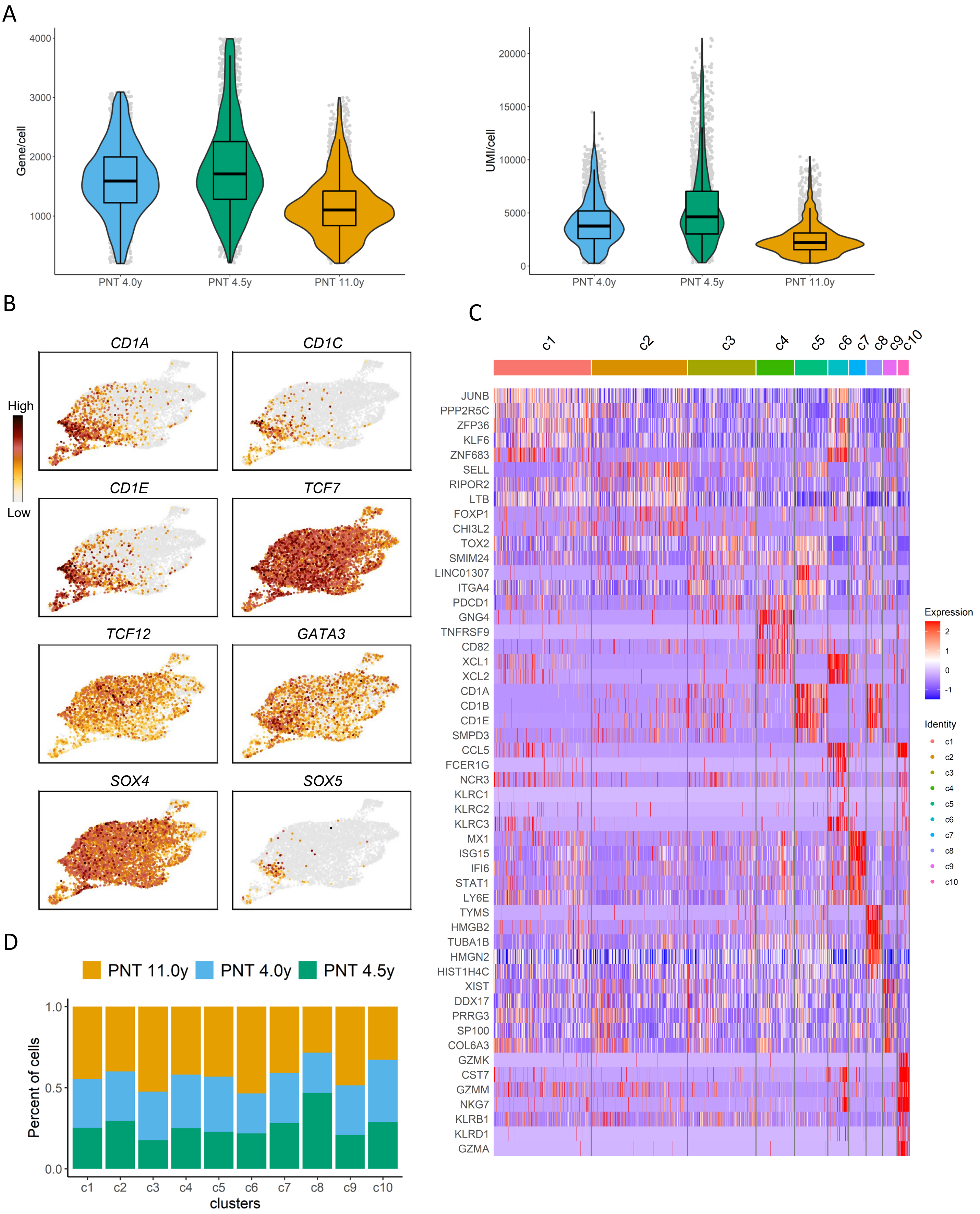

Supplementary Figure 11. (legend: see next page).

Supplementary Figure 11. Pediatric sc γδ thymocyte gene expression data. (A) Quality control (QC) plots of the 3 pediatric thymus sc RNA-seq libraries. **(A, left)** Detected gene number per cell. **(A, right)** Mean number of UMI per cell. The three horizontal lines of the box–whisker plot represent the higher quartile, median, and lower quartile, respectively. The whiskers stretch from each quartile to the maximum or minimum. **(B)** Expression levels of certain genes reported to change during thymocyte maturation. **(C)** Heatmap showing row-scaled Log2 fold change (logFC) expression values of differentially expressed genes (DEGs). List of DGE genes are provided in **Supplementary Data 4**. **(D)** Bar plot displaying individual subject cell distribution across clusters identified in **Fig. 8A.**

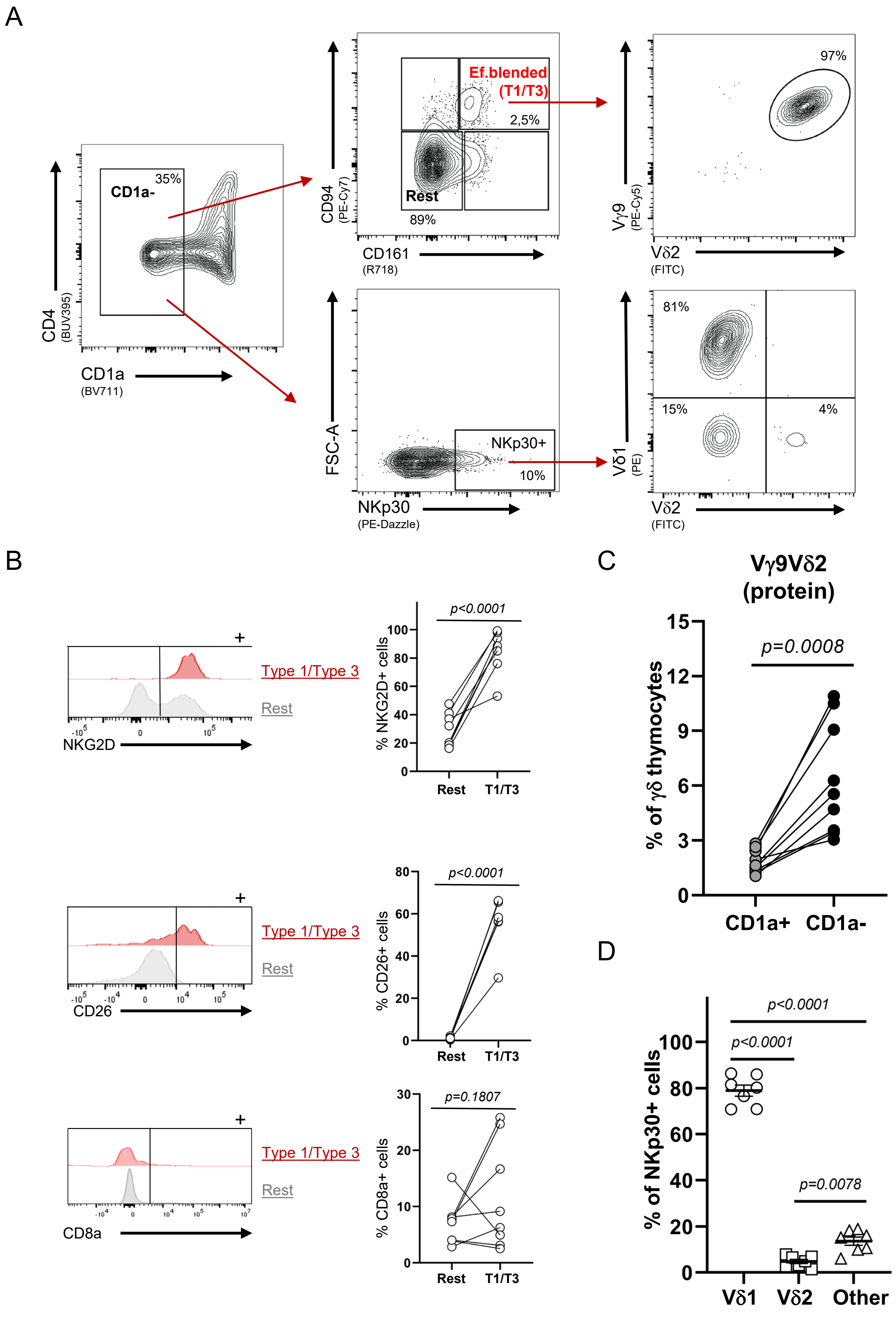

Supplementary Figure 12. (legend: see next page).

Supplementary Figure 12. Protein validation by flow cytometry of the pediatric γδ thymocyte clusters. γδ T cells from 8 post-natal thymy were subjected to flow cytometry analysis to validate key observations described in the sc RNA TCR-seq analysis **(A)** Representative gating strategy used to identify effector blended (T1/T3) and NKp30+ cells, γδ+ cells were gated as CD1a-CD94+CD161$_{hi}$+CD26+ and CD1a-NKp30+ respectively. Cells were further sub-gated to assess the abundance of the distinct γδ subsets in each effector population using Vδ2 and Vγ9 TCR markers for T1/T3 while Vδ1 and Vδ2 TCR markers were used to classify the NKp30+ cells. **(B)** Histogram plots (left) and dot plots (right) representing the abundance of NKG2D+,CD26+ and CD8a+ cells in the distinct clusters.**(C)** Paired dot plot indicating the % of Vγ9Vδ2 thymocytes in the immature (CD1a+) and mature (CD1a-) subsets evaluated at protein level from 8 different subjects. **(D)** Dot plot indicating the Vδ usage of NKp30+ thymocytes identified at protein level from 8 infant subjects. Error bars in **(D)** represent the SEM. Comparisons in **(B,C)** were performed by two-tailed paired t-test. Data in **(D)** was analyzed by Kruskal-Wallis test with Dunn's multiple comparisons test as Post Hoc test. Source data are provided as a Source Data file. In **(B)** "Rest" group is defined as γδ+ CD1a-CD94-CD161- cells and "T1/T3" as γδ+ CD1a-CD94+CD161$_{hi}$+CD26+.

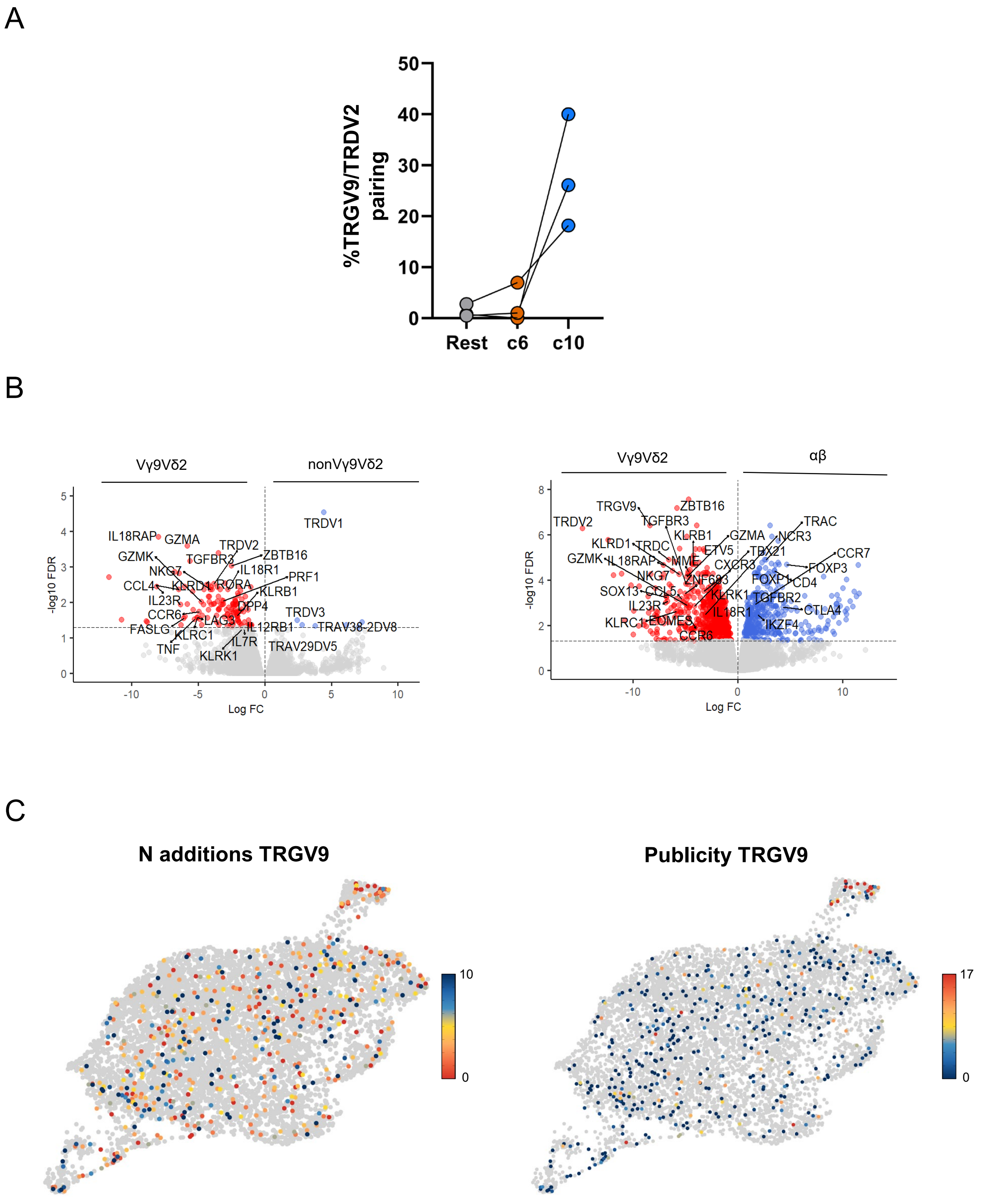

Supplementary Figure 13. The small cluster of pediatric Vγ9Vδ2 thymocytes shows an effector profile and public CDR3γ features. (A) Linked dot plot indicating percentage of Vγ9Vδ2 subset on total γδ T cells (cells with a TRDV and TRGV detected chain) in c6, c9 and the rest of clusters (Other) from the 3 subjects of the pediatric thymus sc dataset. **(B)** Volcano plot illustrating differentially expressed genes obtained from RNA-seq data from Vγ9Vδ2, nonVγ9Vδ2 and αβ T cell subsets sorted from different thymy of infant subjects (n=3, age= 4 months old, 1 and 8 years old; different subjects than the ones of the sc dataset). **(C)** UMAP plots representing number of N additions (left) and levels of publicity (right) of TRGV9-containing CDR3γ sequences detected in the pediatric pediatric thymus sc dataset. Upper limit color scale of N additions plot represents sequences containing 10 or more N additions. Data in **(A)** was compared by Friedman test with Dunn's multiple comparisons test as Post Hoc test. Source data are provided as a Source Data file.

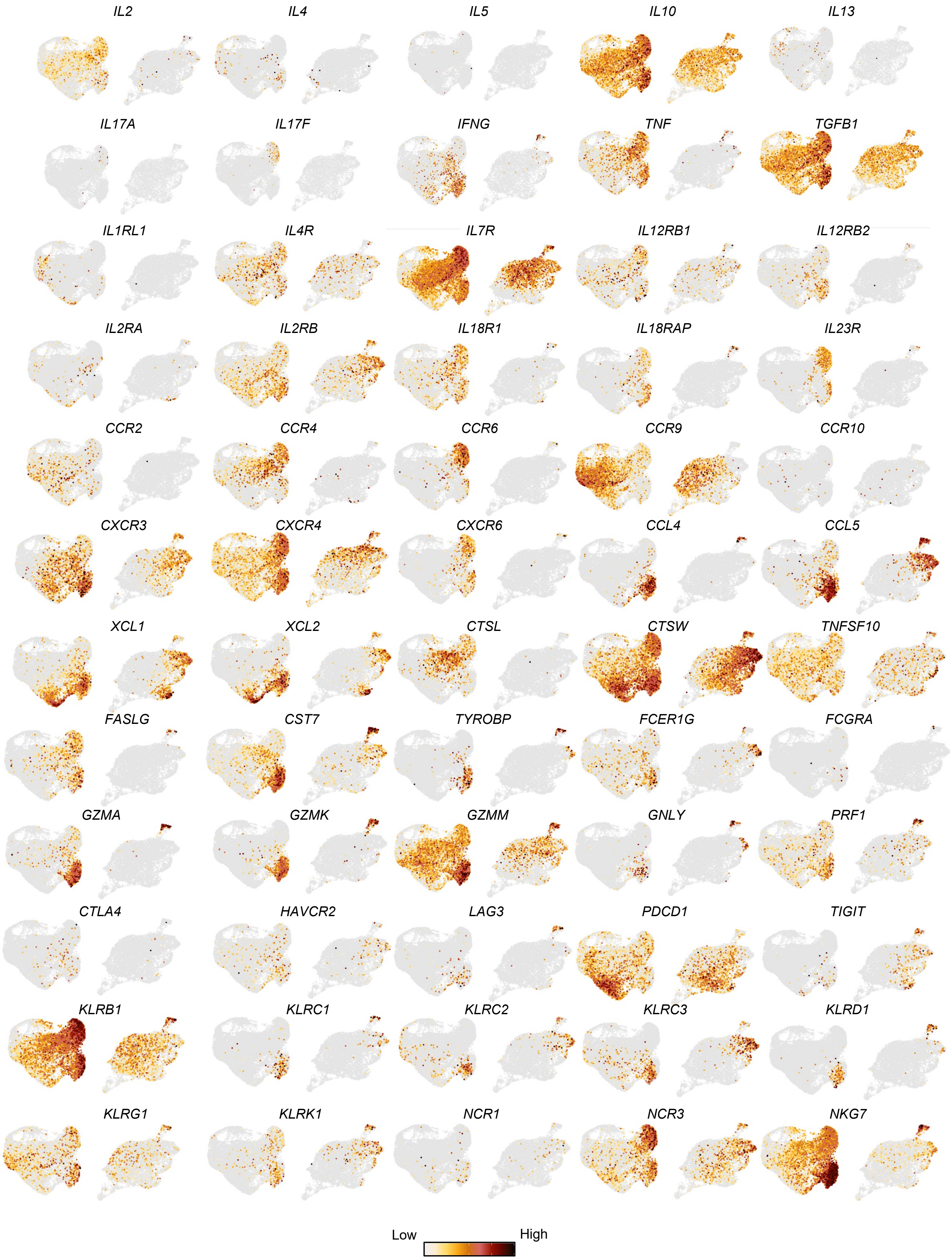
IL2
IL4
IL5
IL10
IL13
IL17A
IL17F
IFNG
TNF
TGFB1
IL1RL1
IL4R
IL7R
IL12RB1
IL12RB2
IL2RA
IL2RB
IL18R1
IL18RAP
IL23R
CCR2
CCR4
CCR6
CCR9
CCR10
CXCR3
CXCR4
CXCR6
CCL4
CCL5
XCL1
XCL2
CTSL
CTSW
TNFSF10
FASLG
CST7
TYROBP
FCER1G
FCGRA
GZMA
GZMK
GZMM
GNLY
PRF1
CTLA4
HAVCR2
LAG3
PDCD1
TIGIT
KLRB1
KLRC1
KLRC2
KLRC3
KLRD1
KLRG1
KLRK1
NCR1
NCR3
NKG7
Low
High

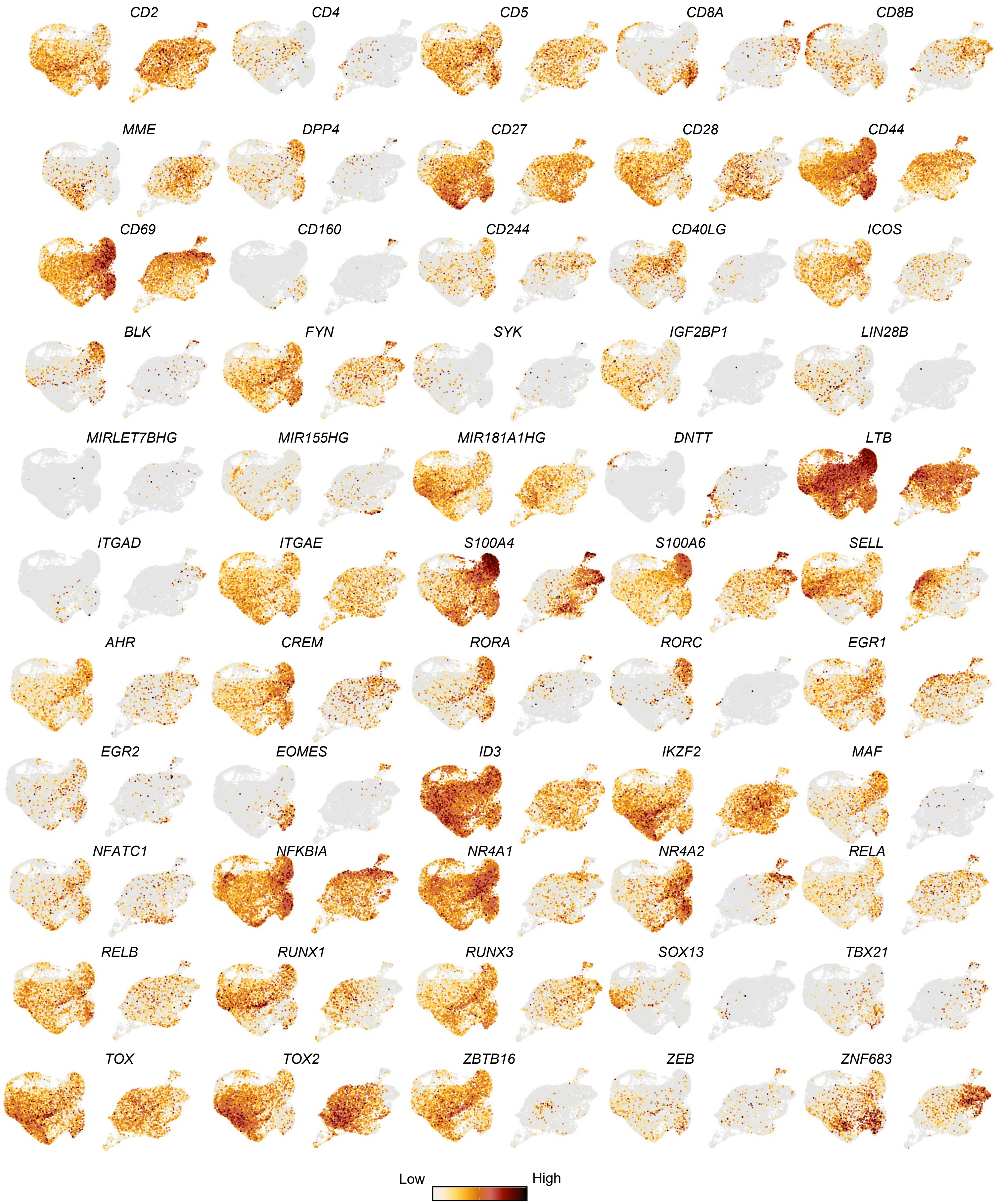

Supplementary Figure 14. (legend: see next page).

Supplementary Figure 14. Comparison of expression levels of selected genes between fetal thymus and post-natal thymus. Expression levels of selected effector molecules, receptors transcription factors and other molecules are projected on a combined UMAP plot generated from fetal (left plot in the combined UMAPs and extracted from **Fig.2A**) and post-natal (right plot in the combined UMAPs and extracted from **Fig.8A**) γδ thymocytes. Color scale (indicating the intensity of the expression of the genes) is the same for both datasets in the combined plots.
