# Supplementary material for: Identification of distinct functional thymic programming of fetal and pediatric human γδ thymocytes via single-cell analysis
Source: Nat Commun. 2022 Oct 4;13:5842. doi: 10.1038/s41467-022-33488-2 (PMC9532436; doi:10.1038/s41467-022-33488-2)
Supplement: Supplementary file 3 — Description of Additional Supplementary Files [file 41467_2022_33488_MOESM3_ESM.pdf]

Supplementary Data 1: List of DGE of the different γδ clusters of the fetal thymus dataset.

Supplementary Data 2: List of DGE of the immature-maturing and effector fetal γδ thymocyte populations.

Supplementary Data 3: List of DGE of the Type 1,3 and 2-like fetal γδ thymocyte populations.

Supplementary Data 4: List of DGE of the different γδ clusters of the pediatrical thymus dataset.

Supplementary Data 5: Lists of markers used to compute module scores.
